# A Clinician's Guide to Cancer-Derived Exosomes: Immune Interactions and Therapeutic Implications

**DOI:** 10.3389/fimmu.2020.01612

**Published:** 2020-07-22

**Authors:** Matthew C. Knox, Jie Ni, Andrej Bece, Joseph Bucci, Yaw Chin, Peter H. Graham, Yong Li

**Affiliations:** ^1^Department of Radiation Oncology, St George Hospital, Kogarah, NSW, Australia; ^2^St George and Sutherland Clinical School, Faculty of Medicine, UNSW Sydney, Kensington, NSW, Australia; ^3^School of Basic Medical Sciences, Zhengzhou University, Henan, China

**Keywords:** exosomes, cancer, immune checkpoint inhibitors, immunotherapy, radiotherapy, cytotoxic chemotherapy, immune modulation

## Abstract

Understanding of the role of immunity in the regulation of cancer growth continues to rapidly increase. This is fuelled by the impressive results yielded in recent years by immune checkpoint inhibitors, which block regulatory pathways to increase immune-mediated cancer destruction. Exosomes are cell-secreted membranous nanoscale vesicles that play important roles in regulating physiological and pathophysiological processes. Cancer-derived exosomes (CDEXs) and their biologically-active cargos have been proven to have varied effects in malignant progression, including the promotion of angiogenesis, metastasis, and favorable microenvironment modification. More recently, there is an increasing appreciation of their role in immune evasion. In addition to CDEXs, there are immune-derived exosomes that facilitate communication between immune cells in the non-malignant setting. Investigation of cancer-mediated mechanisms behind interruption or modification of these normal exosomal pathways may provide further understanding of how malignant immune evasion is accomplished. Accumulating evidence indicates that immune-active CDEXs also have the potential to impact clinical oncological management. Whilst immune checkpoint inhibitors have well-established pharmacologically-targeted pathways involving the immune system, other widely used treatments such as radiation and cytotoxic chemotherapies do not. Thus, investigating exosomes in immunotherapy is important for the development of next-generation combination therapies. In this article, we review the ways in which CDEXs impact individual immune cell types and how this contributes to the development of immune evasion. We discuss the relevance of lymphocytes and myeloid-lineage cells in the control of malignancy. In addition, we highlight the ways that CDEXs and their immune effects can impact current cancer therapies and the resulting clinical implications.

## Introduction

Exosomes are nanoscale extracellular vesicles naturally secreted by most cell types, including cancer cells. They contain a range of bioactive cargos including antigens, proteins, micro-RNAs (miRNAs), and double-stranded DNAs (dsDNAs), facilitating cell-to-cell communication both locally and systemically. Their biological interactions within the field of oncology are undergoing active investigation, with many areas having promising therapeutic implications. Biogenesis, functional cargos, and broad oncological functions of exosomes have previously been reviewed (1, 2).

The anti-tumor immunity generated by exosomes has recently produced much scientific interest. In the most basic sense, exosomes provide a vehicle for tumor antigen delivery to antigen presenting cells (APCs), initiating an adaptive immune response (3, 4). However, investigation has found that other components of exosomal cargo may have immunosuppressive actions, blunting potential anti-cancer effects. As clinical oncology rapidly embraces immunotherapies, understanding and exploiting the role of exosomes in anti-cancer immunity is of great importance.

Currently, the majority of published work is pre-clinical in nature and focused toward basic scientists rather than clinicians. The inherent complexity of exosomal and immunological pre-clinical research makes this published data relatively inaccessible to oncology clinicians. The future involvement of clinicians in the translation of exosomal understanding into effective therapeutic targets is of the utmost importance.

In this review, we aim to briefly summarize this emerging area of research for the benefit of oncology clinicians. We discuss the key mechanisms of cancer-derived exosome (CDEX) mediated immunosuppression for six key immune cells: dendritic cells (DCs), T-lymphocytes, B-lymphocytes, natural killer (NK) cells, macrophages, and myeloid-derived suppressor cells (MDSCs). Further, we provide clinical context around these effects by exploring the ways current cancer therapeutics may impact these pathways.

## Mechanisms of Cancer-Derived Exosome Mediated Immunosuppression

### Dendritic Cells

DCs are potent APCs forming the initial step in adaptive immunity, which plays a central role in anti-cancer immune responses involving T-lymphocyte activation (5). CDEXs have been shown to regulate DC activity via pathways such as DC maturation and apoptosis (6, 7), thus effecting part of the malignant immune evasion process.

DC maturation is an essential component of efficient antigen presentation, upon which immunotherapies involving CTLA-4 checkpoint inhibition rely. DC maturation is reduced in the presence of cancers both *in vitro* and *in vivo* and has been observed across multiple tumor sites (8–10). Further, there is a suggestion that density of DC maturation is potentially an independent prognostic factor in melanoma (11).

CDEXs have demonstrated activity in pathways involved in DC maturation. In one study, human melanoma-derived CDEXs had their cargos evaluated after demonstrating significant inhibition of DC maturation *in vitro* (6). Key proteins isolated from exosomes in this study have previously been shown to be involved in directly influencing maturation, including S100, A8/A9, Annexin A1/A2, and ICAM1 (6, 12). Similarly, prostaglandin-E2 (PGE2) has a demonstrably suppressive effect on DCs and is present in some CDEXs. Human prostate cancer CDEXs purified from cultured cells were found to contain PGE2 and thus upregulated surface CD73 on DCs *in vitro*, resulting in impaired T-lymphocyte activation (13). In these cases, it appears that CDEXs can function to transport directly suppressive molecules to DCs and impact their maturation.

Some other mechanisms for CDEX-mediated induction of DC suppression are not so simple. Liu et al. demonstrated that MyD88 knock-out mice are not impacted by DC inhibition via melanoma-derived CDEXs, while MyD88 wildtype mice are (14). The presence of CDEXs in both groups resulted in upregulation of IL-6 and TNF-α, molecules which are regulated by the MyD88 pathway and can drive DC suppression. MyD88 is an essential molecule in toll-like receptor (TLR) signal transduction and there is evidence that other mechanisms of CDEX-mediated regulation of the TLR pathways also induce DC impairment (15). Thus, pre-existing genetic mutations and transduction pathway defects may be important for exosome-mediated DC suppression.

DC apoptosis is a key mechanism for normal immune regulation. Apoptotic pathways exist as a balance of pro-apoptotic (TRAIL, HLA-DR, TGF-β) and anti-apoptotic (CD40, c-FLIPL, Akt1) pathways (16). Importantly, some factors such as TGF-β can serve both pro- and anti-apoptotic roles in the same cell depending on the cell line, co-stimulatory molecules, and environmental conditions. Cancers are known to secrete non-encapsulated pro-apoptotic molecules which can lead to DC apoptosis, including TGF-β and gangliosides which are secreted from most tumor types (17, 18). These free molecules are depleted in serum via mechanisms such as endocytosis and lysosomal degradation, thus having only limited distant effects (19, 20). However, exosomal contents are protected by the vesicle structure and are not limited in this way, thereby posing the potential for systemic effects.

CDEXs appear capable of inducing DC apoptosis and inhibiting functional antigen presentation. Ning et al. demonstrated increased DC suppression as well as apoptosis following culture with CDEXs isolated from both lung cancer and breast cancer murine cell lines, but did not evaluate the responsible exosomal contents (7).

TGF-β has been widely implicated in immunosuppression due to its ability to impair multiple cell types (including DCs) with respect to function, induced apoptosis, and reduced population expansion (21). TGF-β can induce DC apoptosis both directly and indirectly. Directly, cancer-derived TGF-β concentration has a proven correlation to DC apoptosis in human sentinel lymph node studies *ex vivo* and *in vitro*, without the presence of other contributory cells (17). TGF-β also acts indirectly by first causing upregulation of regulatory T-lymphocytes (discussed later) which then cause apoptosis of DCs (16). Studies demonstrating the presence of TGF-β in CDEXs thus provide a hypothesis for the observed increase in DC apoptosis in the context of malignancy (22). Multiple studies have also demonstrated the presence of Fas-ligand (FasL) and TRAIL in CDEXs, both of which are also involved in DC apoptosis (23).

CDEX-mediated DC suppression has significant detrimental effects on the anti-cancer immune response, with impairment of antigen presentation. This may blunt the efficacy of anti-CTLA-4 therapy (which enhances the priming phase of T-lymphocyte response) as well as immune stimulation resulting from radiation-induced antigen release. Consideration and potential modification of these processes may result in improved clinical outcomes.

### T-Lymphocytes

T-lymphocytes are an essential component of adaptive immunity, fundamentally involved in both the priming and effector processes. Significant evidence exists that cancers often induce immune-evasion through T-lymphocyte modulation and as such, much effort in anti-cancer immune treatments has focused on reducing or harnessing this modulation.

Extensive investigation has focused on exosomal interactions with T-lymphocytes and the immunosuppression that is induced. Pro-apoptotic molecules (such as FasL and galectin-group proteins) are found in CDEXs and are known to induce lymphocyte apoptosis (24–26). However, in addition to inducing apoptosis, many of these molecules can also induce a suppressive phenotype in any surviving cells. Galectin-1 containing exosomes derived from cultured human head and neck squamous cell carcinoma (HNSCC) cells were able to induce CD8^+^ T-lymphocyte suppression *in vitro* following co-incubation (27). This was identified by loss of CD27/CD28 expression and was confirmed by knock-out studies which removed galectin-1 from tumor cells, impairing the previously observed suppressive effect. Further, exposure to the pro-apoptotic TNF and TNFR1/2 found in melanoma-derived exosomes has been shown to contribute to an increase in reactive oxygen signaling (28). This is known to down-regulate T-cell receptor (TCR) expression and thus cause functional impairment.

Another important pathway for T-lymphocyte related immunosuppression is the induction of a suppressive phenotype via upregulation of T-regulatory (Treg) lymphocytes. This effect has been demonstrated by exosomes derived from multiple tumor types including HNSCC, ovarian and sarcoma cell lines (29–33). This exosome-mediated phenotypic change is characterized by upregulation of suppressive molecules classically associated with Treg cells, such as TGF-β, FasL, and CTLA-4 (29). The mechanism for reaching this Treg-predominant suppressed phenotype may involve modified IL-2 reactivity. Whilst IL-2 is typically involved in the stimulation of CD4^+^ T-lymphocyte differentiation into all activated sub-populations, there is evidence that the presence of exosomal TGF-β modulates this process. When exposed to exosomal TGF-β, naive CD4^+^ lymphocytes have reduced activation and differentiation into functional subgroups, with the exception of the FoxP3^+^ Treg subgroup (34). This results in a phenotypically immunosuppressed population of T-lymphocytes.

A further proposed mechanism involves the role of microRNA. Multiple miRNAs including miR-214, miR-155, miR-126, and miR-142 have all been previously implicated in either Treg differentiation or maintenance of the suppressive phenotype (35). The link with exosomes was confirmed experimentally through the demonstration of significantly increased miR-214 expression in CDEXs from multiple human (*ex vivo*) and murine tumor lines (*in vitro*) (31). This was then shown to result in Treg population expansion and functional T-lymphocyte suppression *in vivo* following isolation and injection of exosomes into mice (31). This is an interesting finding given the ongoing interest and investigation into novel gene therapies involving modulation of exosomal miRNAs.

The exact mechanism through which exosomes cause T-lymphocyte modulation remains unclear. Following receptor binding on the membrane surface, immune cells often commence a signaling cascade or effector response via initial receptor internalization. As an example, TCR internalization is an essential component of T-lymphocyte priming. There is some inconsistent evidence that T-lymphocytes may be able to rely on external binding and cell surface signaling only when interacting with CDEXs. This was initially shown by Muller et al. who utilized cultured HNSCC CDEXs in an *in vitro* study to demonstrate that Treg modulation was not associated with exosome internalization (36). This was not replicated in other immune cell populations, as effective endocytosis of the same CDEXs was seen in B-lymphocytes, monocytes and NK cells. The conclusion was that the effect CDEXs have on T-lymphocytes must rely on cell surface signaling alone. However, this was shortly followed by a contradictory study which showed that functional T-lymphocyte suppression was temporally associated with exosome internalization (30). Given the conflicting results, this remains a hypothesis only and requires further investigation.

CDEX-mediated T-lymphocyte suppression has significant therapeutic implications, with biased differentiation of naïve CD4^+^ T-lymphocytes and dominating Treg populations being heavily involved. Whilst detailed understanding exists of many mechanisms involved, methods for therapeutically manipulating and reducing this suppressive effect remain an area requiring ongoing investigation.

### B-Lymphocytes

B-lymphocytes are key cells of the humoral component of adaptive immunity, which facilitates long-term antigen recognition and immune activation. These responses are dependent on the integrity of the antigen-presentation and T-lymphocyte pathways discussed previously. Whilst less significance is often imparted to these cells in clinical oncological research, they serve an important role in cancer control.

B-regulatory (Breg) lymphocytes in the tumor infiltrating lymphocyte (TIL) population are postulated to contribute to the promotion of immune escape and invasion. There is evidence in multiple tumor types of high stromal Breg populations being associated with poorer outcomes (37–43). In addition, increased numbers of peripheral blood Bregs are identified in patients with malignancies (44, 45). CDEXs have been recently implicated in this process. In human hepatocellular carcinoma, co-incubation of isolated CDEXs from cultured cells to a naïve B-lymphocyte population generated expansion of the TIM-1^+^ Breg population *in vitro*, whilst normal hepatocyte-derived exosomes failed to induce this response (43). This effect has also been demonstrated *in vitro* with human esophageal cancer and mycoplasma-infected murine melanoma cell lines (46, 47). Given Breg populations secrete immunomodulatory cytokines such as TGF-β, it is hypothesized that the previously described CDEX-mediated T-lymphocyte suppression may be partially induced by the expansion of a Breg population.

There is also suggestion of a separate mechanism for exosome-mediated immunosuppression involving B-lymphocytes and their antibodies. Peripheral blood samples in patients with pancreatic adenocarcinoma demonstrated an elevated subpopulation of immunoglobulins with bound exosomes (48). Proteomic investigations found these surface-bound exosomes carried antigens consistent with the primary pancreatic cancer. This demonstrates a decoy effect, whereby CDEXs attenuate complement-dependent cytotoxicity by virtue of their binding to circulating autoantibodies (48).

It is clear that CDEXs interact with B-lymphocytes and relevant effector pathways, however the mechanisms are only rudimentarily understood. Further investigation may lead to exploitable therapeutic targets.

### Natural Killer Cells

NK cells are important cytotoxic lymphocytes in innate immunity, analogous to the CD8^+^ cytotoxic T-lymphocyte of the adaptive immune system. They allow an immediate cytotoxic response following recognition of infected or stressed cells in the absence of MHC presentation. Cytotoxicity is regulated by competing stimulatory (e.g., NKG2C/D and CD16) and inhibitory (e.g., NKG2A and KIR) receptors. When bound to ligands which are differentially expressed in stressed and normal cells, these stimulatory receptors induce apoptosis. For this reason, they form an important part of the natural anti-cancer surveillance response.

NK cell numbers and functional cytotoxicity have been extensively shown to be reduced in the presence of malignancy (49–52). This effect partially operates in a similar manner to what has been explained for T-lymphocytes. IL-2 has a central role in the signaling of maturation and the activation of a cytotoxic phenotype. Exposure of NK cells to multiple different human CDEXs containing TGF-β impairs IL-2 reactivity, thus reducing NK cell population expansion and inhibiting cytotoxic function *in vitro* (34). Further, the continued IL-2 induced expansion of the Treg population also directly inhibits NK cytotoxicity, likely through further positive feedback via TGF-β secretion (53, 54).

Instead of relying on MHC-based activation, NK cells possess the NKG2D receptor which is activated by various ligands produced by stressed cells. CDEXs across multiple tumor sites have been demonstrated to harbor these ligands on their surface, with an observed reduction in NK cell membrane NKG2D expression and functional cytotoxicity following co-incubation *in vitro* (22, 55, 56). It has been proposed that a decoy-like effect is responsible, with CDEXs binding to NKG2D and reducing the capacity for cytotoxicity against cancer cells themselves (57, 58). This effect is similar to that described for B-lymphocytes and their antibodies.

Interestingly, there is evidence that hypoxic cancer cells have a unique mechanism for NK suppression. Hypoxic cancer cells have been shown to release phenotypically different miRNA-containing CDEXs and multiple such miRNAs have been implicated in NK suppression (59). Exosomal miR-23a derived from human lung cancer and leukemia cultured cell lines was shown to reduce NK *in vitro* cytotoxicity following co-incubation, likely due to reduced CD107a (LAMP-1) surface expression (60). In addition, the exosomal miRNAs described previously as being implicated in Treg differentiation and maintaining the suppressed phenotype, will also contribute to NK suppression via the same mechanisms detailed above (35).

### Macrophages

Macrophages form part of the innate immune response, acting via phagocytosis to eliminate foreign cells (including cancer cells) and pathogens. They also contribute to the adaptive immune response by acting as APCs in the T-lymphocyte priming process. Tumor-associated macrophages (TAMs) have been identified as forming a large proportion of the stromal cells in tumors. TAMs can be characterized by polarization to either the M1 or M2 phenotype, resulting in broadly tumoricidal and tumorigenic actions, respectively.

CDEXs have been shown to contribute to M2 polarization of TAMs with resulting malignant progression via numerous mechanisms (including impaired anti-tumor immunity) across multiple tumor lines (61, 62). Similar to previously discussed cell types, miRNAs have been heavily implicated in this process across several cancer cell lines (63–66). Human ovarian cancer-derived CDEXs containing miR-222 have been shown to induce STAT-3 (signal transducer and activator of transcription 3) mediated M2 polarization *ex vivo* (63). These CDEXs were then demonstrated to result in tumor progression *in vivo* when introduced into mice. Similar to what was previously described for NK cells, it also appears that hypoxia may play a role in modulating CDEX cargo and thus the induction of a M2 TAM phenotype. Hypoxic pancreatic cancer cells and their CDEXs have been shown to be enriched with miR-301a-3p in a hypoxia inducible factor (HIF) dependent manner (64). When co-incubated with naïve macrophages, these CDEXs induced the M2 phenotype in macrophages via the PTEN/PI3K pathway. The cancer cells themselves subsequently underwent epithelial-mesenchymal transition (EMT) *in vitro*, consistent with an induced metastatic phenotype.

In addition to miRNAs, CDEX-based proteins have also been implicated in TAM polarization. Palmitoylated proteins on the membrane of human breast cancer CDEXs were found to bind to macrophages and resulted in TLR2-mediated activation of the NFκB pathway, which is an important step in polarization to a M2 phenotype (67). This is supported by Bretz et al. who demonstrated that TLR2/4 receptors are necessary for the initiation of signaling via NF-κB and STAT-3 pathways, which can result in the M2 phenotype (68). Further, both human and murine prostate cancer CDEXs were found to contain elevated concentrations of milk-fat globule EGF factor 8 (MFG-E8), a protein which was shown to induce M2 polarization of macrophages *in vitro* following co-incubation (69). Subsequent introduction of an anti-MFG-E8 antibody blunted the initially observed M2 polarization, confirming the influence of MFG-E8 on macrophage polarization (69). The results of this study appear consistent with the suppressive effects MFG-E8 has on inflammatory responses in general.

Given the large population of TAMs in tumor microenvironments, they play significant roles in regulating anti-tumor immunity. CDEX-mediated M2 polarization is clearly a barrier to effective immunotherapeutic strategies and the identification of approaches to reduce this skewed differentiation should be an area of ongoing interest and study.

### Myeloid-Derived Suppressor Cells

MDSCs are a somewhat more recently discovered entity in cancer immunology. They comprise a population of immature myeloid cells which are markedly upregulated in the stroma of cancers and exert powerful immune suppression. MDSCs also contribute to metastasis and tumor progression directly, via several molecular mechanisms. Their importance is highlighted by the correlation between prognosis and both circulating and stromal MDSC concentrations (70–73).

Multiple typical CDEX cargos already discussed in previous sections have been implicated in MDSC differentiation, including TGF-β, PGE2, and heat shock protein 72 (HSP72) (74–76). One of the earlier studies from Xiang et al. demonstrated that CDEXs isolated from cultured mouse breast adenocarcinoma cells contained both PGE2 and TGF-β, resulting in *in vivo* expansion of both tumor and bone marrow MDSC populations (74). This was confirmed by anti-PGE2 and anti-TGF-β antibody studies, which resulted in a marked reduction in both the tumor and bone marrow MDSC populations. Evidence also exists that CDEXs can also mediate or promote the suppressive functions of MDSCs. Chalmin et al. demonstrated TLR2-dependent STAT-3 pathway activation in MDSCs in mice, which was mediated by HSP72 present on the CDEX membrane (75). When STAT-3 was impaired, there was reduced T-lymphocyte immunosuppression and the MDSCs appeared closer in function to those in non-malignant mice.

Again, miRNAs within CDEXs are heavily involved in regulating MDSC populations and immunosuppressive functions, with many different miRNAs implicated (77–81). Human gastric cancer CDEXs have been found to harbor miR-107, which when co-incubated with MDSCs *in vitro* resulted in population expansion and enhanced immunosuppressive function (77). This appeared to be mediated by altered PTEN expression and thus upregulated PI3K pathway activation. As discussed previously, hypoxia-induced modulation of CDEX cargo is again implicated, with miR-21 enriched CDEXs being produced by hypoxic but not normoxic human HNSCC cells (78). These CDEXs were then used in a knockdown study to demonstrate miR-21 dependent MDSC expansion and subsequent T-lymphocyte suppression. Interestingly, this T-lymphocyte suppression was mediated by upregulated MDSC expression of PD-L1, with T-lymphocyte suppression reversed by introduction of anti-PD1/PD-L1 antibodies.

Whilst MDSCs are typically quite short lived (in the order of days), CDEXs may be responsible for enhancing their survival. Exosomes from bone marrow stem cells derived from mice with multiple myeloma were demonstrated to enhance MDSC survival by up to 10-fold *in vitro*, with STAT pathway activation thought to be the primary mechanism (82). Given CDEXs have been shown to upregulate the STAT-3 pathway, it is reasonable to hypothesize that part of the CDEX-induced and MDSC-mediated immunosuppression is facilitated by enhanced MDSC survival.

MDSCs are an important component of the anti-tumor immunity discussion, given the significant degree of upregulation that occurs in the presence of cancers and the wide-ranging immunosuppressive effects they mediate. Further investigation is required into how to best overcome MDSC-induced immunosuppression in the tumor microenvironment to enhance immune therapy outcomes. As discussed above, manipulation of CDEXs may be an attractive approach in achieving this goal.

**Figure 1:**
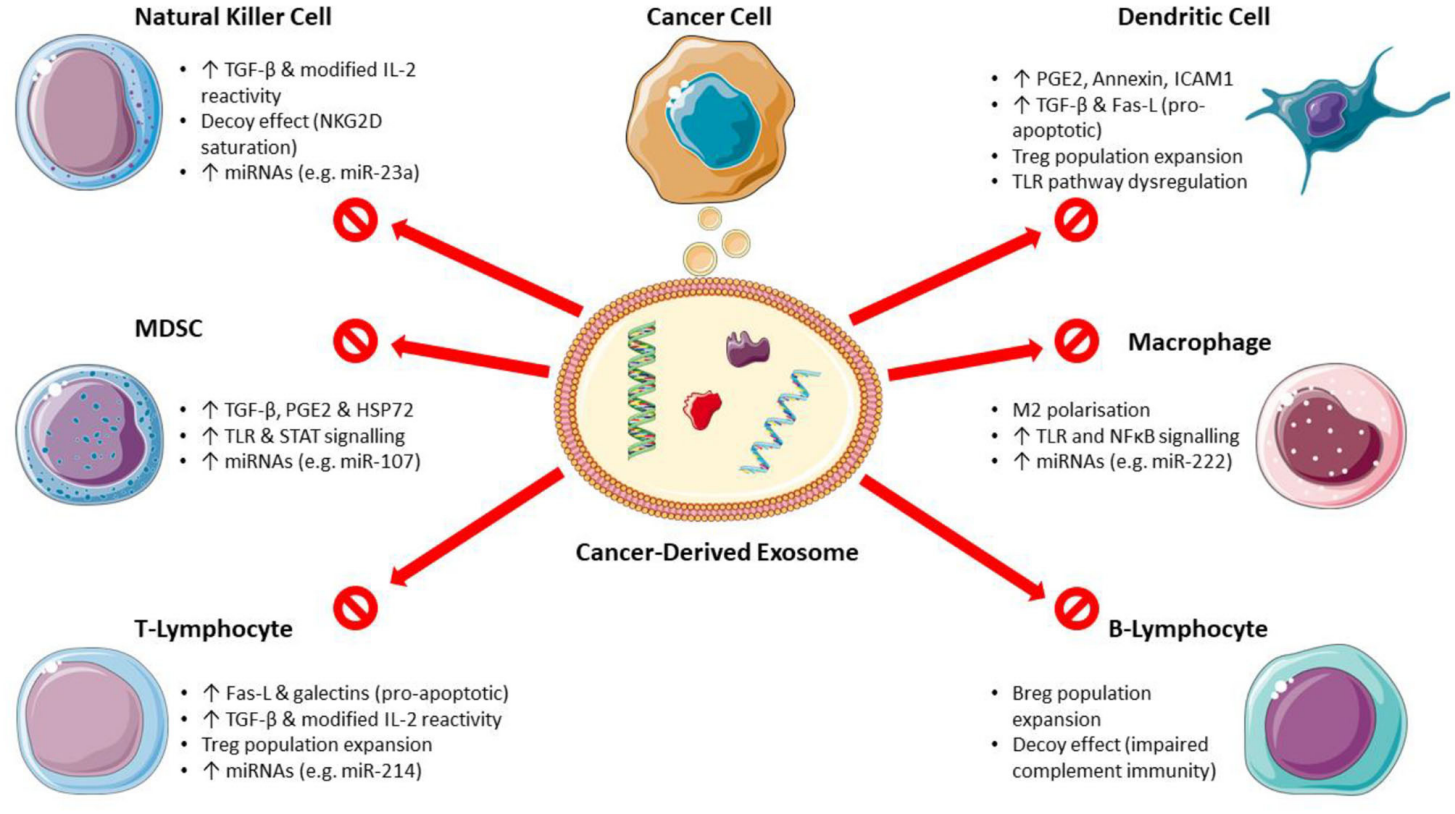
Mechanisms of cancer-derived exosome mediated immunosuppression. (Breg, regulatory B-lymphocyte; Fas-L, Fas ligand; HSP, heat shock protein; ICAM1, intercellular adhesion molecule 1; MDSC, myeloid-derived suppressor cells; miRNA, microRNA; PGE2, prostaglandin E2; STAT, signal transducer and activator of transcription; TGF-β, transforming growth factor-β; TLR, toll-like receptor; Treg, regulatory T-lymphocyte). Images used from Servier Medical Art (https://smart.servier.com) under CC BY v3.0 (https://creativecommons.org/licenses/by/3.0).

The mechanisms of CDEX-mediated immunosuppression on main immune cell types are shown in Figure 1.

## Therapeutic Implications

### Immunotherapies With Immune Checkpoint Inhibitors (ICI)

ICIs such as anti-CTLA-4 and anti-PD-1/PD-L1 antibodies are an increasingly important tool in oncological management. The basic mechanisms of interaction with immune cells are well-understood and previously reviewed, thus we will focus on CDEX interactions only (83, 84).

CDEXs are known to carry PD-L1 within the lipid membrane as well as express the ligand on the membrane surface, meaning CDEX-based PD-L1 may actually exert systemic immunosuppressive effects (85–88). This was demonstrated recently, where CDEX-based PD-L1 suppressed nodal T-lymphocyte activity, encouraged tumor growth at distant sites and was active across multiple murine tumor lines *in vivo* (85). Further, anti-PD-L1 therapy was not as effective in reducing the immunosuppressive effects of exosomal PD-L1, whilst efficacy was maintained against cellular PD-L1. This has also been seen clinically with pre-treatment CDEX-based PD-L1 levels being predictive for response to pembrolizumab, whilst total circulating PD-L1 had a much less significant correlation (86). This effect may play a role in patients who do not respond to anti-PD-1/PD-L1 therapy despite significant tumor PD-L1 expression. Thus, CDEX-based PD-L1 may prove to be a more robust predictive marker for clinical practice.

Changes in PD-L1 concentrations on CDEX membranes during ICI treatment also correlate with efficacy (86, 89). Chen et al. demonstrated that upregulation of PD-L1 expression on human melanoma CDEX membranes during ICI treatment is correlated with improved therapeutic efficacy. Importantly, this observed increase in PD-L1 concentration does not result in a subsequent blunting of anti-tumor responses as may be expected. Because introduction and binding of anti-PD-1 receptor antibodies occurs prior to this treatment-induced ligand upregulation, there are few remaining vacant PD-1 receptors through which PD-L1-mediated immune evasion can result (86).

Furthermore, CDEX-based PD-L1 is predictive for overall cancer burden and prognosis independent of immunotherapy. Patients with HNSCC had CDEXs isolated from the serum and PD-L1 expression was found to be related to disease activity, nodal involvement and TNM staging (UICC TNM Classification of Malignant Tumors) (90). Again, serum PD-L1 levels did not correlate with these findings. A similar effect was also shown by Tucci et al., who demonstrated DC and T-lymphocyte-derived exosomal PD-1 levels were predictive for both overall and progression-free survival in a human population with melanoma (91).

CDEXs enriched with PD-L1 have significant implications on both prognosis and prediction of efficacy of ICI therapy. Whilst high-throughput purification remains difficult and resource intensive, future technological improvements may lead to CDEX-based PD-L1 testing being integrated into routine clinical practice.

### Cellular Immunotherapy

Cellular immunotherapies are a rapidly expanding novel group of therapies, including the well-known chimeric antigen receptor T-cell therapy (CAR-T) and other newer treatments including adoptive NK cell therapy. These therapies involve harvesting the patient's immune cells (cell type dependent on therapy), re-engineering them for cancer-specific activity, *in vitro* clonogenic expansion of the cell population and re-infusion of the cells. In this way, a strong and specific anti-tumor response can be artificially generated, allowing therapeutic results which may persist for long periods of time as the re-engineered cells integrate into the host immune system. At present, research around these therapies has primarily focused on hematological malignancies.

Only very limited research has explored the role of CDEXs in cellular immunotherapy. Principally, a phase-1 clinical trial in patients with relapsed acute myeloid leukemia (AML) demonstrated reduced cytotoxic function and NKG2D expression in the therapeutic adoptive NK cells when incubated *ex vivo* with exosomes isolated from the plasma of AML patients (92). Peripheral blood collected following institution of adoptive NK cell therapy demonstrated a significant *in vivo* reduction in some pro-inflammatory cytotoxic markers (MIP-1a, MIP-1b, and RANTES). Importantly, CDEXs in this study were found to contain elevated levels of TGF-β, CD73, PD-L1, and FasL, all of which have been implicated in CDEX-mediated immunosuppression in the discussions above (92).

The same research group subsequently demonstrated a lack of CDEX internalization into the adoptive NK cells, despite the proven immunosuppression seen above (58). This contrasts with the observed rapid internalization of exosomes by non-therapeutic activated NK cells following co-incubation, implicating a membrane-mediated form of CDEX-induced inhibition for adoptive NK cells.

Whilst the data is clearly immature, this particular study suggests that CDEXs are implicated in treatment resistance and reduced efficacy of cellular immunotherapies. More research in this field is required before any firmer conclusions or mechanistic explanations can be made.

### Radiation Therapy

Radiotherapy is a primary treatment modality in oncological therapy. The observed systemic effects of radiation known as the abscopal effect have long indicated a potential immune interaction, however, only recently have mechanisms been explored.

Ionizing radiation has significant impacts on CDEX synthesis. Through the induction of genomic and physiological stress, post-irradiation exosome stimulation has been demonstrated across multiple malignant cell lines including prostate, HNSCC, breast, and glioma (93–97). This increase in exosome release is also related to p53 mutation status, representing an apparent response to an inability to repair dsDNA damage (93, 94). Of significance, p53 mediated pathways involving increased TSAP6 (transmembrane protein tumor suppressor-activated pathway 6) expression have been widely demonstrated to result in greater quantities of exosomes being released from cancer cells across multiple tumor types (95, 98).

Ionizing radiation can also modify exosomal cargo. One study involving a human HNSCC cell line demonstrated radiation-induced upregulation of proteins such as HMGB-group, HSP-group and FGFR1 proteins (99). This type of protein and miRNA modulation following irradiation has been replicated across other cell lines (100–103). The overall impact is one of a malignant and metastatic phenotype, consistent with damaged cells expressing survival behaviors. Interestingly, observed changes in cargo composition may be dose-related, with a marked difference in miRNA expression seen after irradiation of mice with doses of 0.1 vs. 2 Gy (104).

Radiation-induced neoantigen production secondary to increased tumor mutational burden has been long known. However, this does not directly lead to immune activation which implies that further impacts on immunity are present (4, 105, 106). Immune effects following radiation certainly implicate CDEXs, though multiple mechanisms may exist. Much of the exosomal immune impact following radiation is related to the upregulation of immune-active molecules normally seen in CDEXs. Mrowczynski et al. demonstrated post-irradiation upregulation of multiple CDEX proteins such as TGF-β, and those involved in the Notch and STAT pathways across malignant central nervous system cell lines (100). As detailed previously, TGF-β has a significant role and impacts multiple immune cell types to result in overall suppression of both the innate and adaptive pathways. STAT is involved in signal transduction and can result in the expansion of the immunosuppressive MDSC population, which reduces T-lymphocyte activation (75). Notch is another signal transduction pathway which primarily regulates naïve T-lymphocyte differentiation (107). These pathways are intimately related and together contribute to suppressed phenotypes within the immune system following radiation-induced upregulation.

Damage-associated molecular pattern proteins (DAMPs) within CDEXs have also been heavily implicated. HSP70, HMGB1, and calreticulin are examples of proteins demonstrated to be upregulated within CDEXs following *in vitro* irradiation of melanoma, glioblastoma, and prostate cancer cell lines (101, 108, 109). In general, this group of proteins stimulates immune function via the innate system (both DCs and NKs), which has been demonstrated experimentally. As a specific example, HMGB1 is a transcription factor which is typically released by stressed and necrotic cells, resulting in wide-ranging pro-inflammatory events such as TLR4 and NF-κB activation as well as the release of TNF-α and chemotactic proteins.

Jella et al. have demonstrated the presence of multiple DAMPs in murine melanoma-derived CDEXs, which were induced by *in vitro* irradiation of tumor cells (109). Isolation and then co-culture of these CDEXs with naïve DCs resulted in preferential uptake and activation of DCs (109). Knock-out studies have also shown the importance of these proteins in anti-cancer immune effects following cytotoxic treatments (110, 111). However, this is combated by the concurrent DAMP-mediated activation of MDSCs, which may dampen responses *in vivo* (112). Thus, whilst the exact magnitude of the interaction remains unclear, DAMPs certainly have an important role in post-irradiation immune function.

Radiation-induced exosome release has been implicated in the bystander and abscopal effects, which are rarely seen in practice but are of great interest amongst clinicians. Well-designed studies have demonstrated that CDEXs isolated following irradiation can impact radiation-naïve (bystander) cells with co-incubation (96, 97). Al-Mayah et al. demonstrated dsDNA damage via a comet assay in the bystander group, representing the significance of this effect (96). In contrast, another study demonstrated improved dsDNA break repair and improved survival in bystander cells exposed to irradiated cell CDEXs (97). Whilst the bystander and abscopal effects are often thought to have immune involvement, these studies suggest that at least a portion of the clinical effect may be immune-independent via alternative exosome-mediated pathways.

These observations have led to multiple laboratory and clinical trials combining radiation and immunotherapies. Basic science studies have demonstrated a few key findings: (1) hypofractionated but not single fraction irradiation provides maximal immunogenicity (113), (2) visceral sites are more immunogenic following irradiation than bone (114), and (3) careful timing and sequencing of radiotherapy with ICI therapy is essential (115–117). Unfortunately, early clinical studies investigating combination immunotherapy and radiotherapy were disappointing, in part due to trial designs which did not consider these basic scientific principles (118–120). However, with improved trial design successful results have recently begun to emerge in the literature. PACIFIC was the first successful large phase-3 trial for this treatment approach, demonstrating significantly improved overall survival with the incorporation of durvalumab post-chemoradiation in stage III non-small cell lung cancer (121). Several trials are ongoing or yet to report across a number of tumor sites (122–125).

Thus, it is clear that basic scientific understanding and well-considered study design are integral for successful trials when combining these treatments. Given the important interplay of CDEXs in the immunogenicity of radiotherapy, we would advocate that these should also be subjects of interest and considered as viable biomarkers and targets in future clinical trial design.

### Cytotoxic Chemotherapy

Cytotoxic chemotherapy has been a mainstay of anti-cancer therapy for many decades. Paired with radiation, the treatments demonstrate spatial co-operation with chemotherapy targeting systemic disease. Due to doses used, most chemotherapeutic agents are immunosuppressive as a result of extensive marrow suppression. However, when using modified doses and schedules, there is increasing evidence that cytotoxic chemotherapy may in fact be immunogenic (126, 127).

Increased numbers of CDEXs have been repeatedly demonstrated following administration of cytotoxic chemotherapy (128–132). Furthermore, the constitution of exosomes is different after administration of cytotoxic treatments. Cells appear to demonstrate defensive mechanisms aimed at survival, with the presence of pro-malignant and pro-metastatic exosomal cargos. Myeloma cells treated with bortezemib or melphalan demonstrated marked stimulation of exosome release which contained heparinase, a molecule facilitating extracellular matrix degradation and thus promoting metastasis (128). Breast cancer cells treated with taxanes and anthracyclines demonstrated upregulation of exosomal annexin A6, a molecule associated with metastasis via a broad range of mechanisms (129).

Limited early evidence suggests that the exosomal modulation induced by chemotherapy has a demonstrably immune-active role. One mouse model involving breast adenocarcinoma treated *in vitro* with topotecan demonstrated upregulated secretion of DNA-containing CDEXs as well as STING pathway-dependent (stimulator of interferon genes) DC activation (133). This was replicated *in vitro* with co-incubation of naïve DCs in purified exosomes, where the observed DC activation serves to confirm an exosomal-dependent effect. In contrast, a mixed *in vitro* and *in vivo* study using multiple murine cell lines demonstrated chemotherapy induced immunosuppression. There was an observed increase in HSP70-containing exosomes following administration of cisplatin and 5-fluorouracil *in vitro*, which resulted in MDSC activation following co-incubation and resultant blunting of anti-cancer immune responses *in vivo* (134). With concomitant administration of A8 (a HSP70-inhibitory molecule), less MDSCs were induced *in vitro* and a larger murine *in vivo* anti-cancer immune response resulted. Whilst chemotherapy-induced exosomal modulation of immunity appears to be present, current pre-clinical studies are conflicting with respect to the overall anti-cancer effect. Thus, appreciation of the exact nature of any resulting biological effects is still developing.

Despite the early nature of the evolving evidence, therapeutic approaches are being explored. Combination chemo-immunotherapy has been studied in pre-clinical mouse models and demonstrated promising results (135, 136). One study involving triple-negative breast cancer showed that administration of cisplatin followed by dual anti-PD-1/anti-CTLA-4 immunotherapy resulted in a marked increase in CD8^+^ cytotoxic T-lymphocytes with a decrease in FOXP3^+^ Treg lymphocytes (135). Early clinical trials in humans have also demonstrated positive results. KEYNOTE-189 was the first large phase-3 trial, combining carboplatin/pemetrexed chemotherapy with pembrolizumab in the first-line setting for non-small cell lung cancer, resulting in overall survival and progression-free survival advantages (137). The role of CDEXs in these clinical studies has not been established, though there are likely important functional changes occurring based on the pre-clinical experiences discussed here. Future integration of CDEXs into clinical trials via a translational approach may provide further valuable insights and direction.

Understanding of the role of exosomes in the immunomodulatory effects of chemotherapy is rapidly evolving and under active investigation. Further pre-clinical and clinical studies may assist in clarifying the role of combination therapy and exploring exosomal pathways to improve therapeutic outcomes.

Studies on CDEX-mediated immune interactions with different oncological therapeutic modalities are summarized in Table 1.

**Table 1:** Summary of cancer-derived exosome mediated immune interactions with oncological therapies.

**Treatment**	**Therapeutic interactions**	**Cell lines**	**Study design**	**References**
ICI immunotherapy	CDEX-PD-L1 reduces ICI efficacy	• Human prostate cancer and melanoma • Murine prostate cancer	*in vitro, in vivo* and *ex vivo*	(85)
	• Pre-treatment CDEX-PD-L1 predicts response to ICI	• Human melanoma	*in vivo*	(86)
	• CDEX-PD-L1 changes during treatment correlate to efficacy	• Human melanoma • Human HNSCC	*in vivo* *in vivo*	(86) (89)
	CDEX-PD-L1 is prognostic irrespective of ICI treatment	• Human HNSCC	*in vivo*	(90)
Cellular immunotherapy	CDEX induces reduced adoptive NK cell cytotoxicity (including reduced NKG2D)	• Human acute myeloid leukemia	*in vitro*	(92)
Radiation	Expansion of CDEX population	*(multiple)*		(93–98)
	• p53 regulated response	• Human and murine GBM • Human prostate cancer	*in vitro*	(93, 94)
	Modulation of CDEX cargo			
	• Protein upregulation	• Human HNSCC	*in vitro*	(99)
	• miRNA upregulation	• Human neuroblastoma, GBM and malignant nerve sheath tumor	*in vitro*	(100)
	• Dose dependent changes	• Murine non-malignant splenocytes and bone marrow cells	*in vivo*	(104)
	CDEX-mediated upregulation of regulatory pathways (TGF-β, STAT and Notch)	• Human neuroblastoma, GBM and malignant nerve sheath tumor	*in vitro*	(100)
	Upregulation of DAMP pathways (HSP, HMGB1, calreticulin)	• Human prostate cancer • Human GBM • Murine melanoma	*in vitro* and *in vivo*	(101, 108, 109)
	• Naïve DC activation	• Murine melanoma	*in vitro*	(109)
	• MDSC activation	• Murine renal cancer	*in vivo*	(112)
Cytotoxic chemotherapy	Expansion of CDEX population	*(multiple)*		(128–132)
	Modulation of CDEX cargo			
	• Heparinase (extracellular matrix degradation)	• Human myeloma	*in vitro*	(128)
	• Annexin A6 (pro-metastasis)	• Human breast cancer and melanoma • Murine breast cancer	*in vitro* and *in vivo*	(129)
	CDEX-HSP70 and MDSC activation	• Murine melanoma and lymphoma	*in vitro* and *in vivo*	(134)
	CDEX-DNA mediated STING-dependent DC stimulation	• Murine breast cancer	*in vitro*	(133)

*CDEX, cancer-derived exosome; DAMP, damage associated molecular pattern; DC, dendritic cell; GBM, glioblastoma multiforme; HMGB1, high mobility group box 1 protein; HNSCC, head and neck squamous cell carcinoma; HSP, heat shock protein; ICI, immune checkpoint inhibitors; MDSC, myeloid derived suppressor cell; PD-L1, programmed death ligand 1; STAT, signal transducer and activator of transcription; STING, stimulator of interferon genes*.

## Concluding Remarks

Integrating immunity into cancer therapy continues rapidly with often limited or incomplete basic scientific understanding and foundation. The disappointing results seen in many trials can be at least partially attributed to this fact, with more recent trials incorporating various scientific principles and achieving greater results.

Research into CDEXs has recognized their important role in cancer immune evasion and modulation of natural anti-cancer immune responses. We have shown that in addition to their antigen presentation capability, they directly suppress multiple immune cell types and blunt expected anti-cancer responses. We appreciate that cytotoxic cancer therapies modulate CDEX release resulting in immune interactions, with combination therapies exploiting this.

We suggest several key aspects surrounding CDEX interactions should be considered in future anti-cancer immunity studies: (1) understanding of the kinetics and nature of functional CDEX changes following cytotoxic therapy should contribute to combination therapy design; (2) CDEXs and their functional cargo should begin to be considered prognostic factors, with much ongoing research in this area; (3) CDEX-based PD-L1 should considered a predictive factor for ICIs in addition to tumor PD-L1 expression; and (4) with ongoing refinement and cost-reduction, CDEX assays may provide benefit in regular clinical application.

Oncology clinicians are key drivers in improving care to patients, but without robust scientific understanding and collaboration with scientists, effective translation of laboratory research is difficult. This review provides a comprehensive yet concise summary of CDEX-mediated immunomodulation and its clinical implications for clinicians and encourages further collaborative exploration in this field.

## Author Contributions

MK, JN, and YL reviewed the literature and drafted the manuscript. AB, JB, YC, and PG reviewed and edited the manuscript. All authors contributed to the article and approved the submitted version.

## Conflict of Interest

The authors declare that the research was conducted in the absence of any commercial or financial relationships that could be construed as a potential conflict of interest.

## Abbreviations

APC: antigen presenting cell
Breg: regulatory B-lymphocyte
CDEX: cancer-derived exosome
DAMP: damage associated molecular pattern
DC: dendritic cell
dsDNA: double-stranded DNA
HNSCC: head and neck squamous cell carcinoma
ICI: immune checkpoint inhibitor
MDSC: myeloid-derived suppressor cell
miRNA: microRNA
NK: natural killer cell
PD1: programmed cell death protein 1
PD-L1: programmed death ligand 1
PGE2: prostaglandin E2
STAT: signal transducer and activator of transcription
TAM: tumor-associated macrophage
TGF-β: transforming growth factor β
Treg: regulatory T-lymphocyte.
